# The impact of worsening renal function with elevated B-type natriuretic peptide at discharge on 1-year prognosis in heart failure patients

**DOI:** 10.1038/s41598-020-61404-5

**Published:** 2020-03-10

**Authors:** Toshitaka Okabe, Takehiko Kido, Taro Kimura, Tadayuki Yakushiji, Yu Asukai, Suguru Shimazu, Jumpei Saito, Yuji Oyama, Wataru Igawa, Morio Ono, Seitaro Ebara, Kennosuke Yamashita, Myong Hwa Yamamoto, Kisaki Amemiya, Naoei Isomura, Masahiko Ochiai

**Affiliations:** Showa University Northern Yokohama Hospital Division of Cardiology and Cardiac Catheterization Laboratories 35-1, Chigasaki-Chuo, Tsuzuki, Yokohama 224-8503 Japan

**Keywords:** Biomarkers, Cardiology

## Abstract

There are a few studies about the clinical impacts of plasma B-type natriuretic peptide (BNP) at discharge with the occurrence of worsening renal function (WRF) on mortality in patients with heart failure (HF). We divided total 301 patients with acute decompensated HF into four groups by the median value (278.7 pg/mL) of BNP level at discharge and by the occurrence of WRF. WRF developed in 100 patients (33.2%). Cardiovascular mortality was significantly different between the four groups (P = 0.0002). Patients with WRF and elevated BNP had a higher cardiovascular mortality than patients without WRF and elevated BNP in Cox proportional hazard models (hazard ratio [HR], 10.48; 95% confident interval [95% CI], 1.27–225.53; P = 0.03). Patients with either WRF or elevated BNP did not have an increased risk of cardiovascular mortality compared to patients without WRF and elevated BNP. Regarding HF readmission and cardiovascular mortality, patients with WRF and elevated BNP had the highest risk (HR, 5.17; 95% CI, 2.07–14.30, P = 0.0003) and patients with either WRF or elevated BNP had a higher risk than patients without WRF and elevated BNP. The occurrence of WRF combined with elevated BNP at discharge was associated with increased 1-year cardiovascular mortality and HF readmission.

## Introduction

Heart failure (HF) is a common disease in the world and the prevalence of HF grows with the progression of population aging^1^. Renal dysfunction is highly prevalent in patients with HF and is one of the independent predictors of prognosis in HF patients^2,3^. Worsening renal function (WRF) is one of the major complications that occur in patients with HF. The relationship between WRF and prognosis in patients with HF is still controversial^4–6^. Some studies showed that fluid congestion affected the development of WRF^6,7^. Metra *et al*. suggested that HF patients with WRF and clinical congestion had poorer prognosis than HF patients with either WRF or clinical congestion^8^. However, we often experience subclinical congestion even without clinical sign in patients with HF at discharge. There are a few studies about the association between the hemodynamic congestion/WRF and mortality in patients with HF after discharge. In the present study, we used plasma BNP level at discharge as an indicator of residual congestion in patients with HF because plasma BNP level has a good correlation with high left ventricular end-diastolic pressure that reflects on the hemodynamic congestion, and can easily be measured compared to invasive procedures such as right heart catheter^9^. Therefore, our purpose was to evaluate the association between plasma BNP level at discharge or WRF during hospitalization for HF, and cardiovascular mortality in patients with HF.

## Methods

From March 2010 to July 2016, the medical records of acute decompensated HF patients who admitted to Showa University Northern Yokohama hospital were obtained. Data of systolic and diastolic blood pressure, heart rate, medication, results of echocardiography, history and laboratory values on admission, during hospitalization, and at discharge were collected. Eligible patients were 20 years of age and older, and the diagnosis of HF was based on the criteria of the Framingham study. The HF patients with acute pulmonary embolism, acute coronary syndrome, bradycardia that required pacemaker implantation, or on hemodialysis were excluded. Patients who had plasma B-type natriuretic peptide (BNP) level <100 pg/mL on admission and patients who died during index HF admission were also excluded. A total of 311 patients were included to the cohort after excluding 65 patients without BNP measurement at discharge. Finally, we analyzed 301 patients because 10 patients lost at 1-year follow up after discharge. To assess the relationship between plasma BNP level at discharge or WRF during hospitalization, and outcomes after discharge, we analyzed the only patients whose plasma BNP level at discharge were obtained. We divided the patients into four groups by the median value of plasma BNP level at discharge and the development of WRF during hospitalization. The median BNP level was 278.7 pg/ mL in the present study. The four groups were (1) less than the median BNP level and no occurrence of WRF (W − C−), (2) less than the median BNP level and occurrence of WRF (W + C−), (3) equal or greater than the median BNP level and no occurrence of WRF (W − C+), and (4) equal or greater than the median BNP level and occurrence of WRF (W + C+).

We compared cardiovascular and all-cause mortality, and composite endpoint (cardiovascular mortality and readmission due to worsening HF) within one year after discharge among four groups. We defined the cardiovascular mortality as mortality from ischemic heart disease, arrhythmia, or heart failure. The data of follow-up was obtained by periodic clinical visits, or telephone calls to the patients or their relatives.

The dose of loop diuretics was expressed as furosemide equivalent for some patients who were not received furosemide. The formula for conversion from other loop diuretics to furosemide equivalents was as follows: azosemide 30 mg = furosemide 20 mg^10^. According to previous research for the association between WRF and HF, WRF was defined as an absolute increase in serum creatinine ≥0.3 mg/dL, or a relative increase in serum creatinine of at least 25% from the baseline^5^. Chronic kidney disease (CKD) was defined as estimate glomerular filtration rate (eGFR) <60 ml/min/1.73m^2^. eGFR was calculated from serum creatinine with the Japanese coefficient for the abbreviated Modification of Diet in Renal Disease Study equation^11^.

The present study compiled with the Declaration of Helsinki and the study protocols were approved by the Institutional Review Board of Showa University Northern Yokohama Hospital (approval number 18H055). Waiving the requirement for obtaining written informed consents was allowed by the Institutional Review Board of Showa University Northern Yokohama Hospital, because this study is the retrospective and observational study. The information concerning the present study protocol was announced on the hospital’s homepage to provide the opportunity for the study patients to refuse participation.

## Statistical analysis

Data were analyzed using JMP 14 (SAS Institute, Inc., Cary, NC, USA). Continuous variables were reported as mean ± standard deviation or median ± interquartile range (IQR) which represents the 25th to75th percentiles of the distribution of data. Comparisons between the four groups were performed using ANOVA analysis or the Kruskal-Wallis test for continuous variables, as appropriate. If the different was significant, a Tukey-Kramer test was used to detect which group contributed to the overall statistical significance. Categorical variables were compared between the four groups using Chai-square test or Fisher’s exact test, as appropriate. We then compared bivariate survival curves for all-cause and cardiovascular mortality, and composite endpoint free survival using Kaplan-Meier estimates and tested statistical significance using log-rank test. Univariate and multivariate Cox proportional hazards models were used to evaluate the estimate the hazard ratios (HR) and 95% confidence intervals (CI) for the association between variables and primary/secondary endpoint. Variables with P value < 0.10 in univariate analysis, and those that had been demonstrated to be associated with primary/secondary endpoint in HF patients by previous literature data were included in multivariate Cox proportional hazards models. A two-sided p value < 0.05 was considered to be significant. We created two models for the analyses of cardiovascular and all-cause mortality, and composite endpoint. One included laboratory data on admission and the other included laboratory data at discharge to avoid multicollinearity between laboratory data on admission and at discharge. We forced age and gender in the model. Variables for the models of cardiovascular and all-cause mortality, and composite endpoint were represented below each Table.

## Results

**Figure 1:**
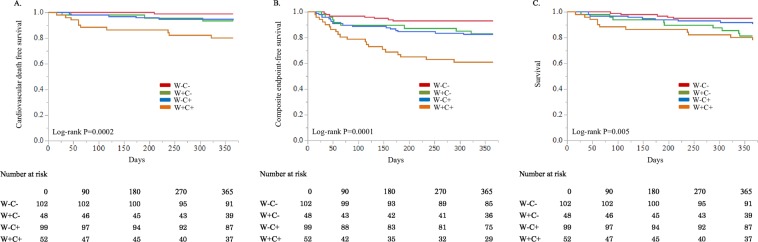
Kaplan-Meier analysis (**A**) Kaplan-Meier cardiovascular death free survival curves for the patients in the four groups. (**B**) Kaplan-Meier composite endpoint free survival curves for the patients in the four groups. (**C**) Kaplan-Meier survival curves for the patients in the four groups.

The patient characteristics are described in Table 1. Among 301 patients enrolled, WRF developed in 100 patients (33.2%). Patients in W − C− group was significantly younger than patients in the others (P = 0.007). Systolic blood pressure was significantly different among the groups (P = 0.04) and tended to be higher in patients in W + C− and W + C+ group than those in W − C+ group. Hypertension was the most common comorbidity in all groups. Left ventricular end-diastolic diameter was smaller in patients in W + C− group than those in W − C+ (P = 0.02) and W + C+ groups (P = 0.04). Left ventricular ejection fraction was higher in patients in W + C− group than those in W − C+ (P = 0.004) and W + C+ groups (P = 0.045). Beta blockers were less frequently used in W + C− group and angiotensin converting enzyme inhibitors/angiotensin 2 receptor blockers (ACEI/ARBs) were less frequently used in W + C+ group (P = 0.08, P = 0.01). Regarding primary endpoint, one (1.0%), three (6.3%), six (6.1%), and 10 (19.2%) patients died from a cardiovascular event in W − C−, W + C−, W − C+, and W + C+ group, respectively. Kaplan-Meier curves for cardiovascular mortality are shown in Fig. 1A. Cardiovascular and all-cause mortality were significantly different between the four groups (P = 0.0002, P = 0.005, respectively). The composite endpoint of cardiovascular mortality and HF readmission was also significantly different between the four groups (6.9%, 16.7%, 17.2%, 38.5%, P < 0.0001) (Fig. 1B). Variables associated with increased cardiovascular mortality were used into multivariate analysis (i.e. age, gender, systolic blood pressure, NYHA functional class, history of HF admission, CKD, left ventricular end-diastolic diameter, left ventricular ejection fraction, beta blocker, ACEI/ARB, aldosterone antagonist, inotropes, and groups of WRF and congestion). In addition, one model included blood urea nitrogen, creatinine, hemoglobin, and sodium on admission and the other included those data at discharge. As shown in Table 2, in all multivariate Cox proportional hazards models, W + C+ was associated with increased cardiovascular mortality (HR, 10.48; 95% CI, 1.27–225.53; P = 0.03 in model 1; HR, 9.51; 95% CI, 1.07–218.13; P = 0.04 in model 2). Patients in W + C− group or those W − C+ group did not have an increased risk of cardiovascular mortality. Regarding the composite endpoint (Table 3), patients in W + C− group, W − C+ group, and W + C+ group were at a significantly higher risk than those in W − C− group. Especially, patient in W + C+ group had the highest hazard ratio (HR, 5.17; 95% CI, 2.07–14.30, P = 0.0003) in the model including laboratory data on admission (Table 3).

**Table 1:** Patient characteristics.

Parameter	W − C− n = 102	W + C− n = 48	W − C+ n = 99	W + C+ n = 52	P value
Age, years	67.6 ± 14.5	72.8 ± 15.1	74.1 ± 13.7*	74.3 ± 16.3*	0.007
Male, n (%)	66 (64.7)	24 (50.0)	67 (67.7)	30 (57.7)	0.18
Heart rate, b.p.m.	98.5 ± 29.6	96.5 ± 23.6	95.9 ± 26.5	98.8 ± 24.7	0.88
Systolic blood pressure, mmHg	139.8 ± 34.0	150.5 ± 36.0	136.4 ± 28.5	148.3 ± 34.4	0.04
Diastolic blood pressure, mmHg	83.7 ± 23.8	89.0 ± 25.7	85.3 ± 20.7	89.3 ± 22.9	0.39
NYHA functional class	3.3 ± 0.7	3.5 ± 0.7	3.4 ± 0.6	3.5 ± 0.6	0.2
History of HF admission, n (%)	15 (14.7)	5 (10.4)	19 (19.2)	18 (34.6)	0.01
HF preserved EF, n (%)	27 (26.5)	16 (33.3)	19 (20.0)	7 (13.5)	0.11
Ischemic heart disease, n (%)	27 (26.5)	11 (22.9)	32 (32.3)	18 (34.6)	0.47
Atrial fibrillation, n (%)	36 (35.3)	15 (31.3)	44 (44.4)	23 (44.2)	0.34
Hypertension, n (%)	66 (64.7)	33 (68.8)	65 (65.7)	42 (80.8)	0.17
Hyperlipidemia, n (%)	46 (45.1)	24 (50.0)	41 (41.4)	23 (44.2)	0.81
Diabetes mellitus, n (%)	38 (37.3)	15 (31.3)	26 (26.3)	18 (34.6)	0.39
CKD, n (%)	44 (43.1)	20 (41.7)	52 (52.5)	30 (57.7)	0.21
LV end-diastolic diameter, mm	54.8 ± 8.9	52.5 ± 9.5	57.2 ± 9.4^†^	57.4 ± 7.9^†^	0.01
LV end-systolic diameter, mm	43.1 ± 11.0	39.5 ± 11.7	46.2 ± 11.4^†^	46.3 ± 9.1^†^	0.002
LVEF, %	43.0 ± 16.6	48.0 ± 14.6	38.8 ± 15.2^†^	39.9 ± 12.9^†^	0.005
Laboratory data on admission					
Albumin, g/dL	3.7 ± 0.5	3.7 ± 0.6	3.6 ± 0.5	3.4 ± 0.5*	0.03
Blood urea nitrogen, mg/dL	21.2 ± 12.3	18.1 ± 6.2	26.9 ± 15.0*^†^	25.0 ± 14.1^†^	0.0003
Creatinine, mg/dL	1.07 ± 0.57	0.91 ± 0.35	1.19 ± 0.57	1.35 ± 0.92*^†^	0.003
eGFR, ml/min/1.73 m^2^	58.2 ± 21.3	62.3 ± 22.6	50.7 ± 18.2^†^	49.9 ± 25.2^†^	0.002
Uric acid, mg/dL	6.9 ± 2.1	6.1 ± 1.9*	7.1 ± 1.9	7.0 ± 2.5	0.06
Sodium, mEq/L	139.5 ± 3.9	140.0 ± 2.9	139.8 ± 4.1	138.7 ± 4.2	0.31
Chloride, mEq/L	105.0 ± 4.2	105.1 ± 4.1	105.9 ± 4.7	105.3 ± 4.5	0.57
Potassium, mEq/L	4.2 ± 0.5	4.0 ± 0.5	4.3 ± 0.7	4.2 ± 0.5	0.07
Hemoglobin, g/dL	12.9 ± 2.4	12.2 ± 2.8	12.3 ± 2.5	11.8 ± 2.5	0.08
Hematocrit, g/dL	38.4 ± 6.6	36.7 ± 7.7	37.1 ± 6.7	35.6 ± 6.8	0.10
BNP, pg/mL	584.5 ± 441.9	461.8 ± 383.9	1181.1 ± 961.6*^†^	1030.1 ± 544.7*^†^	<0.0001
Laboratory data at discharge					
Albumin, g/dL	3.7 ± 0.4	3.6 ± 0.4	3.5 ± 0.5*	3.4 ± 0.4*^†^	0.0001
Blood urea nitrogen, mg/dL	20.0 ± 9.2	23.3 ± 8.9	23.1 ± 10.7	26.9 ± 15.0*	0.003
Creatinine, mg/dL	0.98 ± 0.49	1.06 ± 0.43	1.09 ± 0.44	1.48 ± 1.04*^†‡^	<0.0001
eGFR, ml/min/1.73 m^2^	62.3 ± 22.0	54.9 ± 26.0	54.8 ± 20.9	44.4 ± 21.4*^‡^	<0.0001
Uric acid, mg/dL	6.5 ± 1.9	6.5 ± 1.9	6.7 ± 1.9	7.0 ± 2.2	0.59
Sodium, mEq/L	138.2 ± 3.4	138.5 ± 2.5	139.1 ± 3.1	137.8 ± 4.5	0.11
Chloride, mEq/L	103.5 ± 4.0	104.2 ± 3.1	104.2 ± 4.0	103.8 ± 5.0	0.55
Potassium, mEq/L	4.5 ± 0.4	4.5 ± 0.5	4.4 ± 0.4	4.5 ± 0.6	0.34
Hemoglobin, g/dL	13.0 ± 2.4	12.0 ± 2.4*	12.5 ± 2.3	11.3 ± 2.5*^‡^	0.0005
Hematocrit, %	38.7 ± 6.6	36.1 ± 6.6*	37.5 ± 6.3	34.0 ± 6.8*^‡^	0.0003
BNP, pg/mL	154.5 ± 69.5	135.6 ± 71.2	700.0 ± 437.3*^†^	780.5 ± 536.7*^†^	<0.0001
In-hospital treatment					
Inotropes, n (%)	12 (11.7)	5 (10.4)	10 (10.1)	11 (21.2)	0.28
Intravenous furosemide, n (%)	63 (61.8)	33 (68.8)	63 (63.6)	43 (82.7)	0.04
Dose of IV furosemide, mg/day	20.9 ± 12.9	20.9 ± 12.8	20.0 ± 8.5	21.3 ± 11.0	0.93
Vasodilator, n (%)	64 (62.8)	33 (68.8)	68 (68.7)	38 (73.1)	0.60
Carperitide	58 (56.9)	29 (60.4)	63 (63.6)	38 (73.1)	0.25
Tolvaptan	14 (13.7)	12 (25.0)	20 (20.2)	17 (32.7)	0.048
Medication at discharge					
Beta blockers, n (%)	78 (76.5)	27 (56.3)	73 (73.7)	36 (69.2)	0.08
ACEI/ARBs, n (%)	78 (76.5)	34 (70.8)	70 (70.7)	26 (50.0)	0.01
Loop diuretics, n (%)	71 (69.6)	33 (68.8)	83 (83.8)	42 (80.8)	0.05
Aldosterone antagonists, n (%)	34 (33.3)	20 (41.7)	33 (33.3)	26 (50.0)	0.16

^*^ P < 0.05 compared with W − C− group, ^†^P < 0.05 compared with W + C− group, ^‡^P < 0.05 compared with W − C+ group.

ACEI, angiotensin converting enzyme inhibitor; ARB, angiotensin 2 receptor blocker; BNP, B-type natriuretic peptide; CKD, chronic kidney disease; EF, ejection fraction; eGFR, estimate glomerular filtration rate; HF, heart failure;  IV, intravenous; LV, left ventricular; NYHA, New York Heart Association.

**Table 2:** Multivariate Cox proportional hazards model analysis of cardiovascular mortality.

	Cardiovascular mortality			
	Hazard ratio	95% CI		P value
Model 1				
W + C−	10.29	1.08	232.67	0.04
W − C+	2.88	0.39	58.66	0.32
W + C+	10.48	1.27	225.53	0.03
Reference W − C−				
Model 2				
W + C−	9.25	0.89	222.45	0.06
W − C+	2.40	0.29	53.34	0.45
W + C+	9.51	1.07	218.13	0.04
Reference W − C−				

Model 1 included age, gender, systolic blood pressure, NYHA functional class, history of HF admission, CKD, LV end-diastolic diameter, LVEF, beta blocker, ACEI/ARB, Aldosterone antagonist, inotropes, and groups of WRF and congestion. Model 1 also included blood urea nitrogen, creatinine, sodium, and hemoglobin on admission.

Model 2 included age, gender, systolic blood pressure, NYHA functional class, history of HF admission, CKD, LV end-diastolic diameter, LVEF, beta blocker, ACEI/ARB, Aldosterone antagonist, inotropes, and groups of WRF and congestion. Model 2 also included blood urea nitrogen, creatinine, sodium, and hemoglobin at discharge.

ACEI, angiotensin converting enzyme inhibitor; ARB, angiotensin 2 receptor blocker; CKD, chronic kidney disease; EF, ejection fraction;  HF, heart failure; LV, left ventricular; NYHA, New York Heart Association; WRF, worsening renal function.

**Table 3:** Multivariate Cox proportional hazards model analysis of composite endpoint of cardiovascular death and HF readmission.

	Cardiovascular death and HF readmission			
	Hazard ratio	95% CI		P value
Model 1				
W + C−	3.71	1.24	11.49	0.02
W − C+	3.48	1.40	9.62	0.007
W + C+	5.17	2.07	14.30	0.0003
Reference W − C−				
Model 2				
W + C−	3.69	1.23	11.43	0.02
W − C+	3.56	1.42	9.88	0.006
W + C+	5.21	2.07	14.50	0.0004
Reference W − C−				

Model 1 included age, gender, systolic blood pressure, NYHA functional class, history of HF admission, ischemic heart disease, atrial fibrillation, CKD, LV end-diastolic diameter, LVEF, beta blocker, ACEI/ARB, Aldosterone antagonist, inotropes, and groups of WRF and congestion. Model 1 also included blood urea nitrogen, creatinine, sodium, and hemoglobin on admission.

Model 2 included age, gender, systolic blood pressure, NYHA functional class, history of HF admission, ischemic heart disease, atrial fibrillation, CKD, LV end-diastolic diameter, LVEF, beta blocker, ACEI/ARB, Aldosterone antagonist, inotropes, and groups of WRF and congestion. Model 2 also included blood urea nitrogen, creatinine, sodium, and hemoglobin at discharge.

ACEI, angiotensin converting enzyme inhibitor; ARB, angiotensin 2 receptor blocker; CKD, chronic kidney disease; EF, ejection fraction; HF, heart failure; LV, left ventricular; NYHA, New York Heart Association; WRF, worsening renal function.

However, regarding the all-cause mortality, patients in W + C+ group were not associated with increased all-cause mortality. Only patients in W + C− groups had higher all-cause mortality than others in one of two models (Table 4).

**Table 4:** Multivariate Cox proportional hazards model analysis of all-mortality.

	All-cause mortality			
	Hazard ratio	95% CI		P value
Model 1				
W + C−	3.64	1.06	14.12	0.04
W − C+	0.69	0.17	2.78	0.59
W + C+	2.06	0.52	8.79	0.31
Reference W − C−				
Model 2				
W + C−	3.09	0.85	12.39	0.09
W − C+	0.55	0.14	2.18	0.38
W + C+	1.96	0.52	7.93	0.32
Reference W − C−				

Model 1 included age, gender, heart rate, systolic blood pressure, NYHA functional class, history of HF admission, CKD, LV end-diastolic diameter, LVEF, beta blocker, ACEI/ARB, Aldosterone antagonist, inotropes, and groups of WRF and congestion. Model 1 also included albumin, blood urea nitrogen, creatinine, and hemoglobin on admission.

Model 2 included age, gender, heart rate, systolic blood pressure, NYHA functional class, history of HF admission, CKD, LV end-diastolic diameter, LVEF, beta blocker, ACEI/ARB, Aldosterone antagonist, inotropes, and groups of WRF and congestion. Model 2 also included albumin, blood urea nitrogen, creatinine and hemoglobin at discharge.

ACEI, angiotensin converting enzyme inhibitor; ARB, angiotensin 2 receptor blocker; CKD, chronic kidney disease; EF, ejection fraction; HF, heart failure; LV, left ventricular; NYHA, New York Heart Association; WRF, worsening renal function.

## Discussion

WRF with elevated BNP at discharge was associated with the highest 1-year cardiovascular mortality in the four groups. WRF without elevated BNP at discharge and elevated BNP at discharge without WRF was associated with increased the occurrence of the composite endpoint of cardiovascular mortality and HF readmission. However, neither WRF without elevated BNP at discharge, elevated BNP at discharge without WRF, nor combination of WRF and elevated BNP were associated with increased all-cause mortality. Several reports have demonstrated that the combination of WRF and persistence of clinical congestion was associated with death and HF readmission^8,12,13^. Our findings were consistent with these reports. In the present study, however, cardiovascular and all-cause mortalities (6.4% and 10.9%, respectively) were lower than those in previous large-registries (10.1 to 13.0%, cardiovascular mortality; 18.4 to 23.6%, all-cause mortality)^14,15^. Regarding the composite endpoint, patients with WRF alone and those with elevated BNP alone had higher incidences of the composite endpoint than patients without WRF and elevated BNP – as we usually experience in our daily practice.

Some studies suggested that the persistent congestion in patients with HF was associated with poor outcomes^16,17^. In contrast, the impact of WRF on clinical outcomes in patients with HF is controversial^3,8,18^. We previously reported that WRF was associated with increased cardiovascular mortality. However, WRF was not an independent predictor of cardiovascular mortality in patients with HF^4^. Subsequently, we had recognized that the residual congestion often continued after hospital discharge in HF patients with WRF and assumed that the combination of biomarkers may be useful to predict the prognosis in patients with HF. In the present study, we demonstrated that HF patients with the combination of WRF and elevated BNP had a higher risk of HF readmission and cardiovascular death than those with WRF alone or those with elevated BNP alone.

HF patients with WRF and elevated BNP may have resistance to conventional treatment of HF such as diuretics and this may lead to incomplete de-congestion. In general, creatinine elevation may be caused by several mechanisms. One hypothesis is that increased creatinine may be reflected from rapid fluid removal through HF treatment without tubular injury. In this setting, patients who received aggressive de-congestion treatment tended to have better outcomes regardless the occurrence of WRF^18^. The other is that creatinine elevation may be caused by lack of renal reserve capacity in patients with critical illness^19,20^. Even if serum creatinine or eGFR of these patients have recovered, the transient occurrence of WRF may cause progressive deterioration of kidney tissue and diuretic resistance^21^. It is speculated that sicker HF patients may have greater impaired functional renal reserve by low perfusion and sympathetic nerve activation in addition to fluid rapid removal by HF treatment.

We observed that patients in W + C+ group had a higher hazard ratio for composite endpoint of cardiovascular death or HF readmission than those in W + C− group. Although the diagnosis of WRF and measurement of plasma BNP took place at different times during admission, our findings suggested that aggressive de-congestion can be one of the treatment options to prevent cardiovascular death or HF readmission in HF patients after the occurrence of WRF. One of the mechanisms of residual congestion is diuretic resistance^22^. The additional use of tolvaptan or inhibitors of sodium–glucose cotransporter 2 to loop diuretic may increase the urine output without progression of renal dysfunction^23–25^. However, low perfusion of the kidney due to low cardiac output is still challenging. In patients with residual congestion and WRF because of low cardiac output, novel agents or treatments such as cell therapy are essentially needed to increase the contractility of the myocardium. In the present study, we frequently observed no-cardiovascular death in patients with WRF alone. We may have to take care of comorbidity in these patients such as patients with HF preserved EF^26^.

Creatinine is not a complete indicator of the severity of acute kidney injury. It is recently reported that urine insulin-like growth factor binding protein 7 and tissue inhibitor of metalloproteinase-2 had good accuracy to predict the occurrence of acute kidney injury^27^. Therefore, further research to assess the predictive value of those makers for WRF, congestion, and mortality in HF patients are needed. In addition, there are few data about the prognosis over 1-year in HF patients with WRF and/or congestion. Therefore, further investigations on the long-term prognosis of WRF and/or congestion in patients with HF are warranted.

## Limitation

First, this study is a single center retrospective study in which patients with HF were consecutively enrolled according to our exclusion criteria. Thus, potential selection biases cannot be ruled out. Second, the present study did not include changes in prescriptions in outpatient clinics after discharge for data analysis. We were unable to obtain the data of undiagnosed comorbidities such as cancer, hematologic disorder and respiratory disease. Therefore, unknown confounders might have influenced the results although we performed the multivariate Cox proportional hazard analyses. Third, we could not follow up all patients who relocated or were hospitalized in another hospital. However, follow-up rate was relatively high at 96.8%. Fourth, the definition of WRF in our study might be too sensitive compared to the other criteria (absolute increase in serum creatinine ≥0.5 mg/dL)^28^. Fifth, we divided patients by plasma BNP. Plasma BNP is a good marker of congestion in patients with HF, however our classification may not completely identify the patients with congestion because we used median value for cut-off. Sixth, congestion is not necessary evaluated solely by BNP level in the clinical setting. Seventh, in-hospital treatment of patients with HF was determined by each treating physician. Eighth, we were not able to obtain plasma BNP at discharge from all patients and excluded those without measurement (n = 65). Finally, our findings may not be generally applicable to clinical setting because patient population, geographical region, and ethnic background might be different.

## Conclusions

In this retrospective study, the occurrence of WRF combined with elevated BNP at discharge was associated with increased 1-year cardiovascular mortality and HF readmission.
